# Differentiating Malignant from Benign Pigmented or Non-Pigmented Skin Tumours—A Pilot Study on 3D Hyperspectral Imaging of Complex Skin Surfaces and Convolutional Neural Networks

**DOI:** 10.3390/jcm11071914

**Published:** 2022-03-30

**Authors:** Vivian Lindholm, Anna-Maria Raita-Hakola, Leevi Annala, Mari Salmivuori, Leila Jeskanen, Heikki Saari, Sari Koskenmies, Sari Pitkänen, Ilkka Pölönen, Kirsi Isoherranen, Annamari Ranki

**Affiliations:** 1 Department of Dermatology and Allergology, University of Helsinki and Helsinki University Hospital, 00290 Helsinki, Finland; mari.salmivuori@hus.fi (M.S.); leila.jeskanen@hus.fi (L.J.); sari.koskenmies@hus.fi (S.K.); sari.pitkanen@hus.fi (S.P.); kirsi.isoherranen@hus.fi (K.I.); annamari.ranki@hus.fi (A.R.); 2 Faculty of Information Technology, University of Jyväskylä, 40100 Jyväskylä, Finland; leevi.a.annala@jyu.fi (L.A.); ilkka.polonen@jyu.fi (I.P.); 3 VTT Technical Research Centre of Finland, 02150 Espoo, Finland; heikki.saari@vtt.fi

**Keywords:** biomedical optical imaging, convolutional neural networks, hyperspectral imaging, non-invasive imaging, optical modelling, photometric stereo, skin cancer, skin imaging

## Abstract

Several optical imaging techniques have been developed to ease the burden of skin cancer disease on our health care system. Hyperspectral images can be used to identify biological tissues by their diffuse reflected spectra. In this second part of a three-phase pilot study, we used a novel hand-held SICSURFIS Spectral Imager with an adaptable field of view and target-wise selectable wavelength channels to provide detailed spectral and spatial data for lesions on complex surfaces. The hyperspectral images (33 wavelengths, 477–891 nm) provided photometric data through individually controlled illumination modules, enabling convolutional networks to utilise spectral, spatial, and skin-surface models for the analyses. In total, 42 lesions were studied: 7 melanomas, 13 pigmented and 7 intradermal nevi, 10 basal cell carcinomas, and 5 squamous cell carcinomas. All lesions were excised for histological analyses. A pixel-wise analysis provided map-like images and classified pigmented lesions with a sensitivity of 87% and a specificity of 93%, and 79% and 91%, respectively, for non-pigmented lesions. A majority voting analysis, which provided the most probable lesion diagnosis, diagnosed 41 of 42 lesions correctly. This pilot study indicates that our non-invasive hyperspectral imaging system, which involves shape and depth data analysed by convolutional neural networks, is feasible for differentiating between malignant and benign pigmented and non-pigmented skin tumours, even on complex skin surfaces.

## 1. Introduction

Skin cancers comprise one third of all cancer diagnoses worldwide, and their incidence is continuously increasing [1]. This increase is producing a substantial burden on our health care system, introducing a need for fast and precise diagnostic tools. Dermoscopy, reflectance confocal microscopy (RCM), optical coherence tomography (OCT), and multispectral (MS) imaging are examples of non-invasive imaging tools that have been studied. Among these tools, dermoscopy is the most widely used and accepted, with good evidence for improving diagnostic accuracy [2]. Hyperspectral (HS) imaging is a promising non-invasive imaging option for biomedical and dermatological applications. In contrast to MS imaging, the higher number and width of the spectral channels in HS imaging enables a higher spectral resolution, and thus, recognizes more subtle spectral differences [3]. Longer wavelengths penetrate deeper into the tissue revealing structures and biophysical phenomena, and HS can utilize up to near-infrared (NIR) wavelengths [4].

In dermatological applications, a hyperspectral imaging system (HSI) can identify biological tissues by their diffuse reflected spectra, which depend upon certain tissue chromophores such as melanin, haemoglobin, water, beta-carotene, collagen, and bilirubin [5]. Thus, the advantages of HS imaging offer the unique possibility of visualizing deeper structures (up to 3.5 mm) [4] with a large field of view and fast processing.

In the field of skin tumours, HSI has been used to successfully identify solar field cancerization [6]. HSI can also distinguish lentigo maligna from lentigo maligna melanoma [7], and pigmented basal cell carcinomas (BCCs) from malignant melanomas (MMs) [8], as well as identifying the borders of lentigo maligna [9] and BCCs [10]. HSI has been used successfully in melanoma screening [11,12,13,14] and the diagnosis of skin lesions [15]. However, there are few HSI studies on non-pigmented lesions [6,10], and in several studies, the diagnosis of supposedly benign lesions was not histologically confirmed, possibly biasing the results. Based on previous HSI studies, one of the major challenges is the complex surface topography and tomography of certain areas of the body, such as the shoulder, nose, ear, and other parts of the face.

HS imaging has advances and challenges. An HS image consists of a stack of frames. Each frame represents the intensity of a different wavelength of light, and each pixel (px) has a spectrum. The spectral data have both spectral and spatial domains, which provide detailed information on the target. Another major challenge for HS image processing is the large amount of data. An imager can capture tens or hundreds of frames, which can lead to the Hughes phenomenon, whereby the accuracy gradually increases as the number of dimensions increases, but decreases after a certain number of dimensions is reached [16], as well as producing redundancy among the samples [17]. These challenges can be avoided computationally, for instance, by employing common feature extraction methods. In this study, our solution was to customise the imager to capture only the necessary wavelengths. By selecting the wavelength channels and corresponding light-emitting diodes (LEDs) to represent the spectral absorption peaks of tissue chromophores from visible (VIS) to NIR light and using common reflectance calculations, the HS image contained the main diffuse tissue reflectance spectrums, thus providing a multidimensional view of a lesion with depth information. In this way, we employed a computationally effective solution since the amount of captured data and pre-processing could be limited.

**Figure 1:**
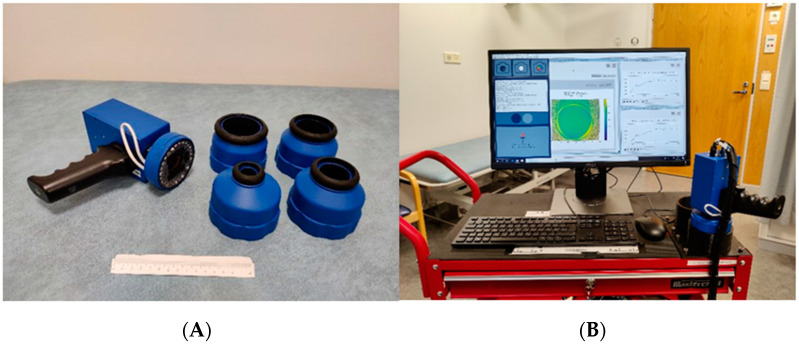
The SICSURFIS imager, light-emitting diode (LED) module, and stray light protection cones (**A**). The imaging setup and software (**B**). Image source [18].

This study represents the second stage in our three-stage pilot project to introduce and clinically test a new concept for skin cancer diagnosis. The core of the study is the SICSURFIS Spectral Imager, which is a compact, hand-held, piezo-actuated metallic mirror Fabry–Pérot interferometer (FPI)-based hyperspectral imager suitable for complex skin surfaces, as described in detail elsewhere [18]. The imager has integrated LED-based illumination, which is set to special lightning angles for photometric stereo imaging. Each LED and FPI position are individually controllable. Since the FPI is the spectral separator, the captured wavelengths can be selected by the software to match the LED illumination. The imager’s field of view is adaptable based on the different sizes of specially designed light protection cones, which block unwanted light and, through their soft silicon collars, adapt to complex skin surfaces more easily than the imagers used in previous studies. The system’s photometric stereo imaging provides skin-surface models, which, combined with the spectral and spatial domains, introduce more information on the analysed complex surfaces to the machine learning model. Another advantage of the SICSURFIS Spectral Imager is its feasibility: the hand-held device is small and light and can be used by one person without assistance (Figure 1).

In this study, we captured HS images of pigmented and non-pigmented malignant and clinically reminiscent benign skin lesions using the novel SICSURFIS Spectral Imager. After imaging, the lesions were excised for histological analyses. The data were pre-processed with photometric stereo imaging methods (see the detailed explanation in Hakola et al. [18]), which provided skin-surface models for each of the captured wavebands. Besides the skin-surface models, the raw data were calculated from albedo images.

As a classification and delineation method, we used a specially designed convolutional neural network (CNN) that utilises spectral and spatial information (albedos) with the skin-surface model. Previous studies have estimated CNNs for diagnosing skin tumours based on plain red-green-blue (RGB) or dermoscopy pictures as accurately as an experienced dermatologist—at times even outperforming the human experts [19,20,21,22,23]. We calculated both the pixel-wise and majority voting classification results.

The aim of this pilot study was to compare the discriminatory capacity of combined HSI and CNN analysis for pigmented and non-pigmented, malignant, and benign skin tumours with similar appearances. As this combined analysis includes more data on the shape and depth of the lesion than previous studies with HSI [18], our hypothesis is that high resolution photometric stereo imaging and specific depth data for each wavelength will improve the classification and delineation results compared to previous studies, and that complex skin surfaces will no longer be a major challenge.

## 2. Materials and Methods

### 2.1. The Clinical Study

The clinical study was performed in February and March, 2020, at the Dermatology Outpatient Clinic, Helsinki University Hospital (HUS). The volunteering patients were recruited in an unselected fashion from patients attending the dermatology outpatient clinic due to diagnosed or suspected skin cancer, or from patients that wished to have their likely benign nevus removed. All patients provided their written informed consent. The study protocol followed the Declaration of Helsinki and was approved by the Ethical Review Board of HUS.

The physicians (V.L., K.I.) and the research nurse (J.Y.) captured images of the lesions in vivo with the HSI, as well as by digital and dermoscopy imaging, and subsequently biopsied or removed all lesions for the histological analyses. The histological analysis of each sample was performed by an experienced dermatopathologist (L.J.) at the dermatopathology laboratory of the Skin and Allergy Hospital, HUS. In total, 42 patients with 54 lesions were recruited. Of these, 6 lesions were excluded due to imaging artefacts (e.g., imaged with a stray light protection cone that was too small) and 6 lesions were excluded due to other histology (lentigo, seborrheic keratosis, neurofibroma, pyogenic granuloma, or carcinoma in situ). Thus, the final analyses included 42 lesions from 33 patients. As pigmented lesions, we studied 7 MMs and 13 benign pigmented nevi (PN); and as non-pigmented lesions, we studied 10 BCCs, 5 squamous cell carcinomas (SCCs), and 7 benign intradermal (ID) nevi. Pigmented nevi included both combined and junctional nevi. ID nevi were clinically non-pigmented.

### 2.2. Patient Demographics and Lesion Characteristics

Patient demographics and lesion characteristics are listed in Table 1. Seventeen patients had a history of one or several skin cancers, and six had a history of one or several other cancers. Four patients were on immunosuppressive medication, among whom one was an organ transplant patient. Thirteen (31%) of the lesions were located on the head or neck, including three lesions on particularly complex sites (the ear, eyelid, and corner of the eye). The Breslow thicknesses of the SCCs varied between 1.1 and 5.2 mm, and that of the melanomas varied between 0.2 and 1.6 mm. All melanomas were of the superficial spreading type. The melanoma with a Breslow thickness 1.6 mm had a diameter of 28 mm and surrounding satellite lesions.

### 2.3. The SICSURFIS Hyperspectral Imager

The SICSURFIS Spectral Imager (VTT Technical Research Centre of Finland, Espoo, Finland) prototype used in this study is composed of a hand-held Piezo-actuated metallic mirror FPI hyperspectral imager, an RGB sensor, and an LED light source. The hand-held device weighs only 890 g. The light source has three series of intentionally selected nine LEDs, emitting light from white to 940 nm. The LEDs can be tilted at specific angles to enable photometric stereo imaging. Each LED and FPI position are individually controllable. Since the FPI is the spectral separator, the captured wavelengths can be selected to match the LED illumination by the software. To enable the imaging of complex skin surfaces, the imager has four stray light protection cones (Figure 1) that block unwanted light and adjust the imager on the skin. The stray light protection cones are different sizes (3.1–26.4 cm^2^), and thus, offer the imager an adaptable field of view for different-sized lesions [18]. The soft silicon collars adapt to the shape the skin and provide comfort. Detailed block diagrams and details of the imager and its calibration processes can be found in [18].

The imager is connected to a computer via Cube View software [24] (University of Jyväskylä, Spectral Imaging Laboratory [25]). The software is pre-set to capture 33 wavebands with wavelengths from visible to near infrared light (477–891 nm) [18]. The user interface is designed for an effortless workflow, allowing the user to concentrate on the patient. With one click, the imager captures three sets of raw frames (in total, six HS images) of one lesion with different light directions, providing spectral data for the three-dimensional skin-surface models and the albedos. The imager has a pixel resolution of 1 px ≈ 24 × 24 µm. The tissue penetration depth depends upon the wavelength; for the wavelengths used in this work, the penetration depth was 0–6 mm [26]. Ease of use was considered when developing the software, which guides the user to capture dark and white references, followed by images, using three different LED light and waveband combinations with one click.

### 2.4. Data Pre-Processing

The three sets of raw frames, each including 33 wavelengths, were pre-processed twice. The two stages were raw image pre-processing and machine learning pre-processing. The detailed information and mathematical formulas for this process can be found in [18].

### 2.5. Raw Data Pre-Processing

**Figure 2:**
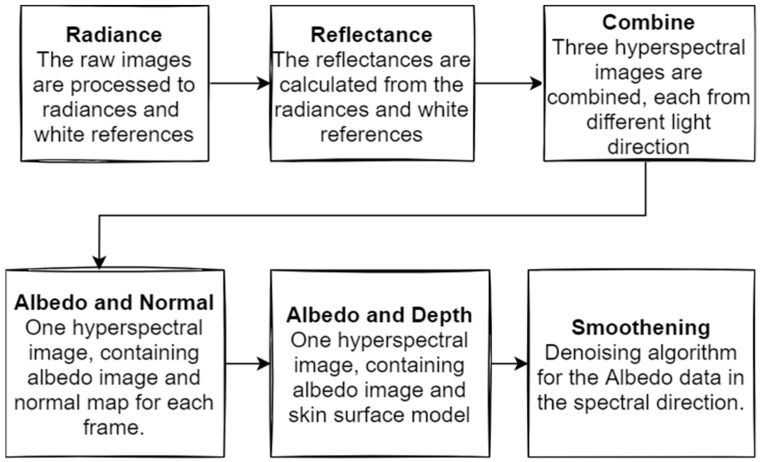
Raw-image pre-processing. The detailed process and mathematical formulas can be found in [18].

The SICSURFIS imager is a high-quality *wavelength scan* HS imager with a collection speed similar to that of a *snapshot scan imager* [3], which captures the wavelengths frame by frame. The raw image pre-processing was conducted as shown in Figure 2. We obtained six raw HS images for each lesion. The VIS and visible and near-infrared (VNIR) channels were captured under three different angles of light. These images were calculated to determine their radiances and white references. The radiances and white references provided six reflectance HS images. The different light angles were combined into three HS images. The albedos and normals were calculated based on these images, forming one HS cube per lesion. The depth maps (the skin-surface model) were calculated from these cubes, and, after smoothening, one HS cube per lesion was formed. Each cube contained the albedo images and skin-surface models for each of the 33 captured wavelengths.

**Figure 3:**
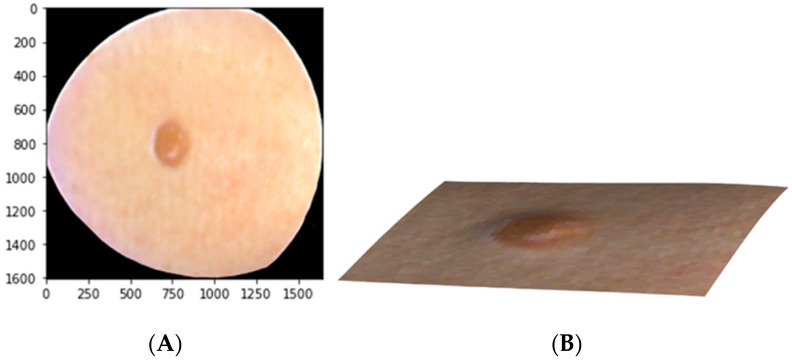
Example of the data after the raw image pre-processing. Reconstruction of an ID nevus using red-green-blue (RGB) from the albedo images (**A**) and its skin-surface model (**B**).

Since an HS image (HS cube) is a stack of frames, and the size of one frame was 1605 × 1640 px, the size of the whole processed HS cube was 1605 × 1640 × 66 px after the raw image pre-processing phase. Here, the first 33 of 66 pixels represented the skin-surface albedo images and the last 33 pixels were the respective skin-surface models. An “RGB image” reconstruction from the albedo images consisting of three wavelength channels is illustrated in Figure 3 on the left. On the right is a reconstruction of the skin-surface model for the same ID.

### 2.6. Machine Learning Pre-Processing

**Figure 4:**
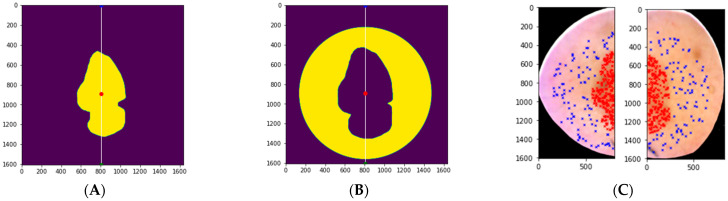
The ground truth with the image sliced through the middle (white line); training data on the left and test data on the right (**A**). The healthy skin mask (**B**). The training and test data. Blue represents the healthy skin pixels; red represents the lesion pixels (**C**). The size of the original lesion annotation was minimized to a lesion binary map of 30 pixels and similarly enlarged to a healthy skin mask. A 60-pixel margin at the lesion border was applied where no pixels were selected.

A physician (V.L.) manually annotated the RGB images (i.e., marked the areas of tumour and healthy skin based on a histological diagnosis). The annotations were digitised to binary form and formed the ground truth (Figure 4). We then created healthy skin masks, which were necessary for selecting the healthy skin pixels around the lesion. Our first SICSURFIS study highlighted that the skin-surface models had the best quality in the middle of the images. We also noted some uncertainly labelled pixels in the lesion border areas, which may have influenced the results [18]. To overcome this, we created an algorithm that provided an annular boundary around the lesions to leave out low-quality areas at the periphery of the images. A 60-px-wide margin at the lesion border was also left out of the analyses to reduce bias from the annotations.

After finishing the masks, we selected the most significant skin-surface frame, which was the 575 nm wavelength. Each HS image consisted of 33 albedo images and the 575 nm skin-surface frame. Selection was based on our first pilot study [18]. The data were normalised between 0 and 1, and the possible infinity and NaN values were set to 0.

Figure 4 presents the training and test pixel selection, and the masks. Before windowing and selecting the training and test pixels, the images were vertically sliced through the middle of the lesions. The left side of each HS image was taken as the training image and the right side as the test image.

For each training and test image, 250 lesion and 100 healthy skin pixels were selected randomly. These pixels were the middle pixels of 30 × 30 pixel windows with 34 channels, which were selected using a rolling-window algorithm [18]. After data selection, the training set’s windowed pixels were balanced using the imbalanced learn library random over-sampling method [27] and augmented with vertical and horizontal flipping. The training set for classification of the pigmented lesions (MM, PN, and healthy skin) included 29,136 px, the validation set included 1400 px, and the test set included 7000 windowed px. The non-pigmented (BCC, SCC, ID, and healthy skin) training set encompassed 31,168 px, the validation set totalled 1540 px, and the test set totalled 7700 px. The machine learning models were tested via pixel-wise classification with whole HS images (1605 × 1640 × 34 px), which were pre-processed similarly with the test and training data. The windowed pixels from whole HS images were used for predicting the classification and confidence maps for each lesion image and for the majority voting analysis. Detailed information on the applied pre-processing algorithms has been described elsewhere [18].

### 2.7. Data Analysis

**Figure 5:**
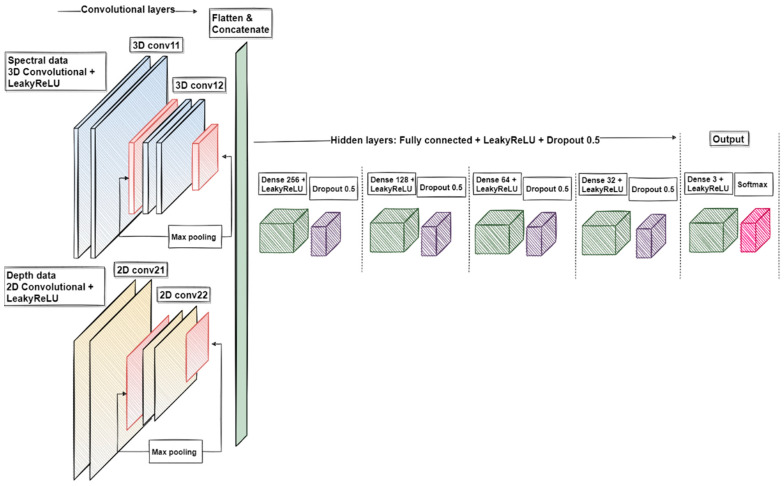
A visualization of the convolutional neural network. The 3D convolutional layers were used with the albedo images, and the 2D convolutional layers processed the skin-surface model. The outputs were concatenated, flattened, and used as input for the hidden layers. Depending on the examination, the output layer was 3- or 4-class classification (dense layer). Image source [18].

The pre-processed training and validation data were analysed at the University of Jyväskylä using CNN models. We used two models, a three-class classifier (MM, PN, and healthy skin) for the pigmented lesions and a four-class classifier (BCC, SCC, ID, and healthy skin) for the non-pigmented lesions. Both models had similar structures, wherein the 3D and 2D layers were used to utilize the spectral, spatial, and depth information from the data. The CNN 3D layer extracted features from the albedo images, and the 2D layer extracted features from the skin-surface models. The CNN construction included the LeakyReLu activation function and max-pooling layers as shown in Figure 5.

After feature extraction, the 2D and 3D layer results were flattened, concatenated, and used as input for the hidden layers. Depending on the examination, the final output dense layer provided the results for three- or four-class classification. The model was trained via the Adam optimizer with default parameters using the categorical cross-entry loss function.

With both test setups (i.e., the three- and four-class classifiers), the results were obtained both pixel-wise and for the whole HS image. The pixel-wise results were conducted from randomly selected windowed test data containing the lesion types and healthy skin samples. The whole windowed HS images were classified to produce the classification confident and classification maps for visual evaluation. Since the whole HS images also contained pixels outside the imager’s field of view (the black area seen in image C, Figure 4), the overall accuracy results were biased by these outlier pixels. Therefore, we performed a majority voting test for the whole image classification results. With the original lesion binary maps delineated by a dermatologist and confirmed via histology, we selected all pixels that were annotated as lesion pixels from the right side of the image (sliced vertically as in the training and test data, Figure 4). Then, we calculated the number of lesion pixels for each lesion type. By assuming that there was only one type of lesion pixel per lesion, we re-classified the images to the majority class. That way, majority voting provided one primary diagnosis for each lesion based on the most common diagnosis for the lesion pixels in the test data.

We provided accuracy reports for both the pixel-wise analysis and majority voting. The analysis included sensitivity, specificity, positive predictive values (PPVs), and F1-score measurements, which were provided by Scikit-Learn metrics [28]. The results were completed via confusion matrices.

The study was implemented with Scikit-Learn [28], Scikit-Image [27], SciPy [29], and Tensorflow Python libraries [30]. Computing was performed using a Linux GPU server, 1 × Tesla P100, x86_64. The HSI and computational analyses are described in detail in a previous article by Raita-Hakola et al. [18].

## 3. Results

### 3.1. Classification Results for Pigmented Lesions

**Figure 6:**
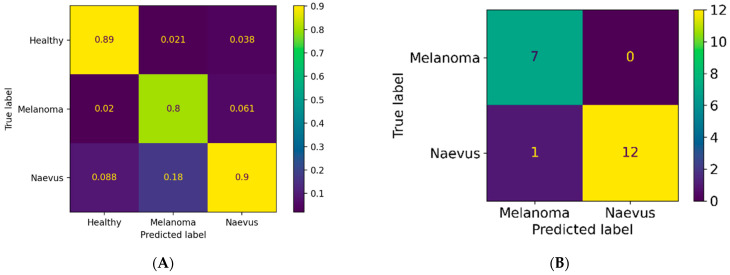
Confusion matrices presenting positive prediction values (PPVs) for pigmented lesions in pixel-wise (**A**) and majority voting analyses (**B**). Melanoma = malignant melanoma, naevus = pigmented nevus.

**Figure 7:**
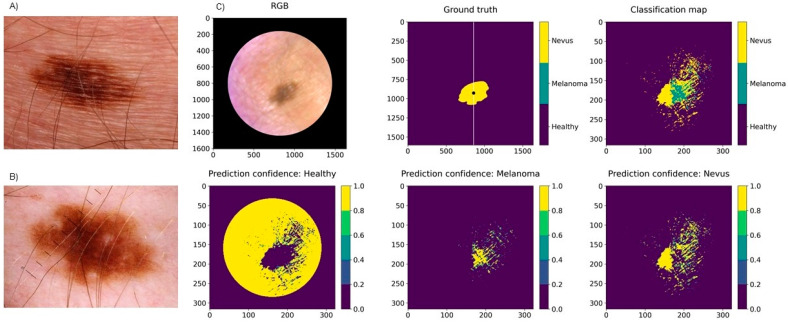
Clinical (**A**) and dermoscopy images (**B**) and a classification map (**C**) of a 10 mm low-grade dysplastic compound nevus of the chest, clinically and histologically diagnosed as a benign nevus but classified as a melanoma by the SICSURFIS system. The test data (right half of the image) included pixels classified as both melanoma and nevus. However, according to the majority voting analysis, there were more melanoma pixels. Light reflection caused probable artefacts in the surrounding area of the lesion.

In total, 20 pigmented lesions, 7 MMs, and 13 benign PNs were studied. The pixel-wise classification for pigmented lesions reached a weighted sensitivity of 87%, a specificity of 93%, and a PPV of 87%. In majority voting, the weighted sensitivity was 95%, specificity was 97%, and PPV was 96%. None of the melanomas were classified as nevus, but one low-grade dysplastic benign compound nevus was classified as melanoma by the majority voting test. Figure 6 presents the PPVs for the separate diagnoses by each analysis method. A representative example of a pixel-wise classification map for a pigmented lesion is illustrated in Figure 7.

### 3.2. Classification Results for Non-Pigmented Lesions

**Figure 8:**
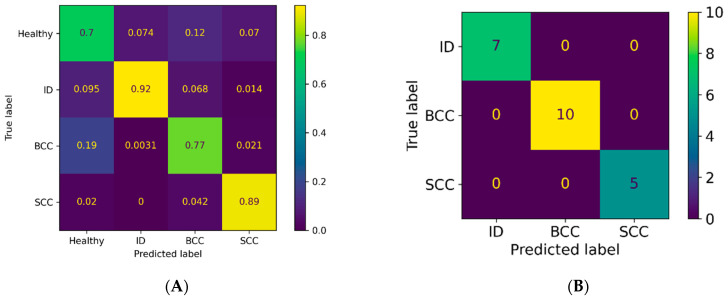
Confusion matrices presenting the PPVs for non-pigmented lesions in the (**A**) pixel-wise and (**B**) majority voting analyses.

**Figure 9:**
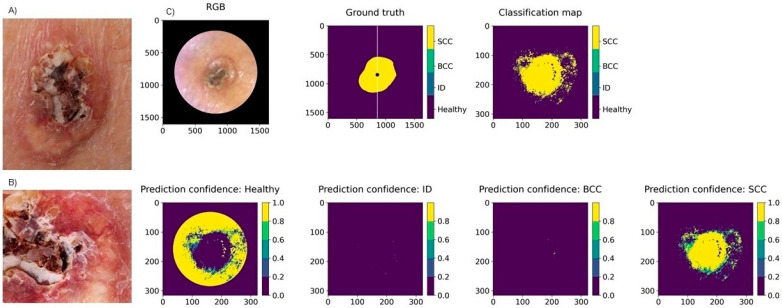
Clinical (**A**) and dermoscopy image (**B**) and the classification map (**C**) of a 15 mm SCC on the leg. Clinically, this lesion could be either a BCC or a SCC, but it was correctly classified as a SCC by the system. The SICSURFIS system delineated the lesion accurately, although it was surrounded by some probable imaging artefacts caused by the uneven skin colour of the healthy skin.

The pixel-wise classification for non-pigmented lesions (22 lesions: 10 BCCs, 5 SCCs, and 7 benign IDs) reached a weighted sensitivity of 79%, a specificity of 91%, and a PPV of 80%. In the majority voting analysis, the weighted sensitivity was 100%, specificity was 100%, and PPV was 100%. Thus, all lesions were classified correctly by majority voting. Figure 8 presents the PPVs for the separate diagnoses by each analysis method, and Figure 9 illustrates a pixel-wise classification map of a non-pigmented lesion.

## 4. Discussion

We achieved a sensitivity of 87% and a specificity of 93% for recognising melanoma from pigmented nevi and healthy skin with a pixel-wise analysis, and even higher results using majority voting (95% and 97%, respectively). Two previous studies on melanoma recognition combined HSI with deep learning. Hirano et al. studied 619 lesions and classified them utilizing GoogLeNet pretrained with ImageNet [12]. However, the results were unsatisfactory, achieving a sensitivity of 72% and a specificity of 81% after data augmentation, likely due to the reduction of the wavelengths used from 84 to 3. Three wavelengths are considered more comparable to digital or multispectral, rather than hyperspectral, imaging. Kato et al. similarly utilized a pretrained GoogLeNet to analyse 619 lesions [13]. The authors used automated analyses with transfer learning and reached somewhat higher classification results based on two analysis methods with sensitivities of 80% and 77% and specificities of 82% and 82% after data augmentation. Our results are more accurate but remain in line with those of Kato et al. Thus, it seems that the results obtained via HS imaging are repeatable.

In previous studies on the recognition of pigmented lesions with HSI that did not utilize machine learning, Christensen et al. studied 202 skin lesions and reached a sensitivity of 97% for malignant lesions and a specificity of 42% for benign lesions [11]. Three other well-powered studies reached sensitivities of 88–97% and specificities of 87–100% [14,31,32]. However, none of these previous studies histologically confirmed the diagnosis of supposedly benign lesions [11,12,13,14,31,32], and thus, the results are not directly comparable with those obtained for histologically verified lesions. In the present study, all lesions were confirmed histologically, and only healthy skin was assessed by clinical inspection alone.

CNN analyses of digital or dermoscope images have achieved similar, or even greater, accuracy for melanoma recognition than that achieved by dermatologists [19,20,21,23]. For instance, in a study by Haenssle et al. on dermoscopy images, 58 dermatologists reached a sensitivity of 87% and a specificity of 71%, whereas the CNN achieved a higher specificity of 83% [20]. In a meta-analysis of 22 melanoma recognition-studies, dermoscopy-CNN achieved a sensitivity of 90% and a specificity of 74% [21]. For MS imaging with computer-aided diagnosis, the values for sensitivity and specificity based on 15 studies were 93% and 44%, respectively, demonstrating that MS imaging is often limited by low specificity values [33]. In a recent meta-analysis of melanoma detection via RCM, the pooled sensitivity and specificity were 92% and 70%, respectively [34]. Two studies on OCT reached sensitivities of 74–89% and specificities of 61–92% [35,36]. Thus, the results of our HS analyses are strong and offer high specificity values compared to other analysis methods. In the studies by Pezzini et al. and Gamblicher et al. on RCM and OCT [34,36], trained experts were required for the analyses.

For the detection of non-pigmented or non-melanoma skin cancer (NMSC) with HSI, studies are rare. Our results seem promising with a sensitivity of 79% and a specificity of 91% for separating BCC, SCC, ID, and healthy skin, considering that non-pigmented lesions are more difficult than pigmented lesions to diagnose via dermoscopy. For the majority voting analysis, all non-pigmented lesions were classified correctly (sensitivity and specificity of 100%). A meta-analysis of the dermoscopy of BCC reached a sensitivity of 93% with a fixed specificity of 80%, but no conclusions could be drawn on the dermoscopy of SCCs [37]. CNN has been compared with medical personnel for recognizing malignant pigmented and non-pigmented lesions from dermoscopic and digital images, and it achieved a higher sensitivity of 81% compared to human raters (78%) [22]. Our HSI-CNN system achieved a higher accuracy compared to the aforementioned study by Tschandl et al. [22]. HSI images include wavelengths of NIR in addition to the VIS light used solely by digital and dermoscopy imaging; thus, some tissue chromophore-specific information that is not visible to the human eye may be obtained by HS imaging.

For non-pigmented skin lesions, a meta-analysis of RCM including four studies on BCC reached a pooled sensitivity of 76% and a specificity of 95% [38]. Another meta-analysis of three studies utilizing OCT reached a sensitivity of 95% and a specificity of 77% [39]. Notably, in these studies, machine learning was not used for interpretation, and a high-level of expertise is required for the use of RCM and OCT. Studies on MS imaging of NMSC are rare [40,41], making it impossible to draw conclusions on this technique’s feasibility.

The PPV can be used to describe the performance of a diagnostic imaging tool. The pixel-wise analyses of pigmented lesions had a higher PPV for nevus (90%) than for melanoma (80%) (Figure 6). Thus, most of the pixels classified as nevus were true nevus pixels (90%), but a high number of pixels classified as melanoma were true nevus pixels (18%). Additionally, many healthy skin or nevus pixels were classified as melanoma—in total, 20.1% (false positives). Only 8.1% of true melanoma pixels were misclassified (false negatives), which is acceptable for a diagnostic tool that is used to recognize a malignant condition.

For the single case where a 10 mm low-grade dysplastic combined nevus was classified falsely as a melanoma by majority voting (Figure 7), the lesion’s more strongly pigmented parts were classified as melanoma, while the less strongly pigmented parts were classified as nevus. In the pixel-wise map, about as many pixels were classified as melanoma as nevus. Dysplastic nevi are atypical both clinically and histologically and can thus have similar features to melanomas. Clinically speaking, it is better that majority voting classified a lesion as a melanoma, as it would be more serious to misdiagnose a melanoma as a nevus. As only a small portion of the lesion was evaluated by histology (we analysed eight cuts), and the whole lesion was analysed by HSI, it could be speculated that HSI may recognize areas of early melanoma growth that were overlooked by histology.

For the non-pigmented lesions, the PPV was higher for ID (92%) and SCC (89%) than BCC (77%), as a high number of true BCC pixels were classified as healthy skin (19%) (Figure 8). In our study, four of the ten BCCs belonged to the superficial or partly superficial subtypes. Tumours of this subtype can be ill-defined at the tumour border, and small tumour islands can be spread out among healthy skin at the lateral tumour border. As these islands are not detectable with the naked eye, parts of the actual BCC can be annotated falsely as healthy skin, impairing the results of the HSI-CNN. In contrast, nodular BCC, SCC, and ID usually have a clear and well-defined tumour border.

Because of the small number of samples, the CNN in this study was trained on one half of a lesion, and the other half of the same lesion was used for the analyses. This approach may have positively influenced the results, as the training and test data were similar. We noticed that colour changes in the healthy skin (reflections, uneven skin tone, hair, and nipples) were occasionally classified as lesions by the CNN and not as healthy skin. This result is understandable, as we did not have a class for features such as hair, whose colour and structure differ from healthy skin. Another limitation of this study when considering biology is that the CNN should have also been trained on actinic keratoses and in situ carcinomas, which are precursors of SCC and can surround SCCs on sun-damaged skin.

The SICSURFIS Imager can capture lesions of different sizes that are located on difficult sites, although the risk of imaging artefacts is higher for lesions on complex sites. The smallest protection cone is ideal when capturing small lesions on complex sites. With such a cone, the correct distance to the lens is more easily maintained, enabling the image to remain focused. However, when using the smallest cone, the amount of healthy skin surrounding the lesion was found to be insufficient. When using the larger cones for these locations, stray light was able to pass the protection cone and cause artefacts. Using a broader soft ring or cloth around the protective cone could decrease the risk for artefacts, and images of exclusively healthy skin would provide the additional data required for healthy skin. Some artefacts could have been caused by movement of the imager during imaging, as the process required up to 20 s. A faster imaging process could thus enhance the image quality.

The strengths of this study include the histological verification of all lesions and the multi-classification of lesions into different diagnosis categories, not just malignant or benign. The SICSURFIS imager has a high resolution and was developed for photometric stereo imaging. Moreover, the feasibility of both the imager and the software has improved. Unlike other studies [14], we included lesions from complex sites. Additionally, we used a CNN (not only indexed limit values) for the analyses and employed both spatial and spectral data. The CNN used in this pilot study could be further trained and applied in larger-scale studies.

Based on these results and previous studies on HSI, it seems that HSI could be used not only to aid in the diagnosis of pigmented and non-pigmented lesions but also to form a classification map of lesions that could be used to delineate lesions more accurately, and to indicate different diagnoses or parts with deeper invasion in a lesion, thus detecting the most informative biopsy site of a lesion [10,42]. Although even experienced dermatologists can mistake malignant skin lesions as benign, this diagnostic aid could be even more helpful for non-dermatologists. Contrary to many other imaging techniques, HSI does not require training or histological knowledge for the user [5]. Accurate delineation could, moreover, reduce the need for re-excisions of skin tumours. Reducing the number of biopsies and re-excisions would save costs and thus be important for the treatment of the increasing number of skin cancer cases in the future.

In conclusion, the results of this pilot study with 42 pigmented and non-pigmented lesions indicate that the novel SICSURFIS HSI-CNN system with shape and depth data can reliably aid in the differentiation of malignant and benign skin lesions, even on complex skin surfaces. All lesions, except one, were diagnosed correctly by the majority voting analyses. However, the results must still be validated by larger-scale studies, which are anticipated to reveal the advantages of our 3D spectral imaging system.

## Figures and Tables

**Table 1:** Patient demographics and lesion characteristics (*n*, %).

**PATIENTS**	**33**	**LESIONS**	**42**
**Mean age**	**68**	**Mean diameter**	**10.3 mm (2–30 mm)**
**Males**	**16 (48%)**	**Diagnosis**	
**Females**	**17 (52%)**	**Pigmented lesions:**	
**Fitzpatrick skin type**		**MM**	**7 (17%)**
I	9 (27%)	Superficial spreading MM	7 (17%)
II	12 (36%)	**PN**	**13 (31%)**
III	12 (36%)	Junctional nevi	5 (12%)
**History of skin cancer**	**17 (52%)**	Compound nevi	8 (19%)
BCC	8 (24%)	High-grade dysplastic PN	2 (5%)
MM	7 (21%)	Low-grade dysplastic PN	3 (7%)
SCC	2 (6%)	Nevus recurrence	1 (2%)
BCC + MM	3 (9%)	**Non-pigmented lesions**:	
**History of other cancers**	**6 (18%)**	**BCC**	**10 (24%)**
Breast	3 (9%)	Nodular	6 (14%)
GI	2 (6%)	Nodular + superficial	3 (7%)
Prostate	2 (6%)	Superficial	1 (2%)
Blood	1 (3%)	**SCC**	**5 (12%)**
**Immunosuppression**	**4 (12%)**	**ID**	**7 (17%)**
**Radiation therapy**	**3 (9%)**	**Location:**	
**Multiple nevus syndrome**	**2 (6%)**	Head/neck	13 (31%)
**Dysplastic nevi**	**6 (18%)**	Torso	21 (50%)
**Family history of skin cancer**	**4 (12%)**	Upper extremities	2 (5%)
**Multiple nevus syndrome in the family**	**4 (12%)**	Lower extremities	6 (14%)

BCC = basal cell carcinoma, GI = gastrointestinal, ID = intradermal nevus, MM = malignant melanoma, PN = pigment nevus, SCC = squamous cell carcinoma.

## Data Availability

The data presented in this study are not publicly available or available upon request due to ethical and privacy reasons.
